# A Case of Pulmonary MALToma Camouflaged Within Invasive Pulmonary Aspergillosis

**DOI:** 10.7759/cureus.53256

**Published:** 2024-01-30

**Authors:** Gurumurthy Thilagavathy, RK Sasankh, Arul Sekary, Niranjan Prabhu SS

**Affiliations:** 1 Pulmonology, Vijaya Hospital, Chennai, IND; 2 Cardiothoracic Surgery, Vijaya Hospital, Chennai, IND

**Keywords:** tuberculosis associated obstructive pulmonary disease, upper lobe lobectomy, haemoptysis, invasive aspergillosis, pulmonary maltoma

## Abstract

A 59-year-old non-smoking male, with a known case of COPD (chronic obstructive pulmonary disease), treated pulmonary tuberculosis with Category 1 antitubercular drugs (six-month regimen) and was admitted with repeated bouts of moderate haemoptysis (~60 mL/day) for three days. The patient had a history of self-limiting occasional mild haemoptysis (~20 mL) over three years. An HRCT chest revealed a left upper lobe fibro-cavitary lesion with an intracavitary mass (air crescent sign), adjacent pleural thickening and fibrosis. Bronchoalveolar lavage (BAL) was positive for galactomannan and negative for *Mycobacterium* tuberculosis GeneXpert®. With the above clinical factors, host factors, and microbiological factors, the case was diagnosed as ‘probable’ invasive pulmonary aspergillosis and was treated with voriconazole. However, given relapsing haemoptysis despite adequate antifungal treatment, a left upper lobectomy was done. The resected left upper lobe specimen culture demonstrated *Aspergillus fumigatus* with histopathology confirming hyphae invading lung tissues confirming ‘proven’ invasive aspergillosis. Resected tissue also showed florid lymphoid tissue hyperplasia with Immunohistochemistry confirming the presence of a peculiar malignancy; MALT lymphoma/MALToma in the resected lobe. The association of a rare malignancy such as MALToma with invasive pulmonary aspergilloma (IPA) has been identified and reported for the first time. This could be because of a chronic inflammatory reaction elicited by the *Aspergillus *antigen. Long-standing fibro-cavitary disease and aspergillosis are partners in crime, augmenting the damages inflicted by one another. In such a scenario, early surgical intervention may be warranted if haemoptysis is moderate to severe or relapsing, following conservative medical management. Surgical resection may lead to the identification of unexpected diseases as in our case.

## Introduction

Aspergillosis is the most common fungal infection of the lungs with a wide spectrum of presentation depending on the host immunity and underlying structural lung damage [1]. In the literature, pulmonary malignancies are rarely reported in the setting of aspergillosis. Here, we present one of the rarest primary pulmonary malignancies: pulmonary MALToma for the first time in conjunction with invasive pulmonary aspergillosis (IPA).

## Case presentation

**Figure 1:**
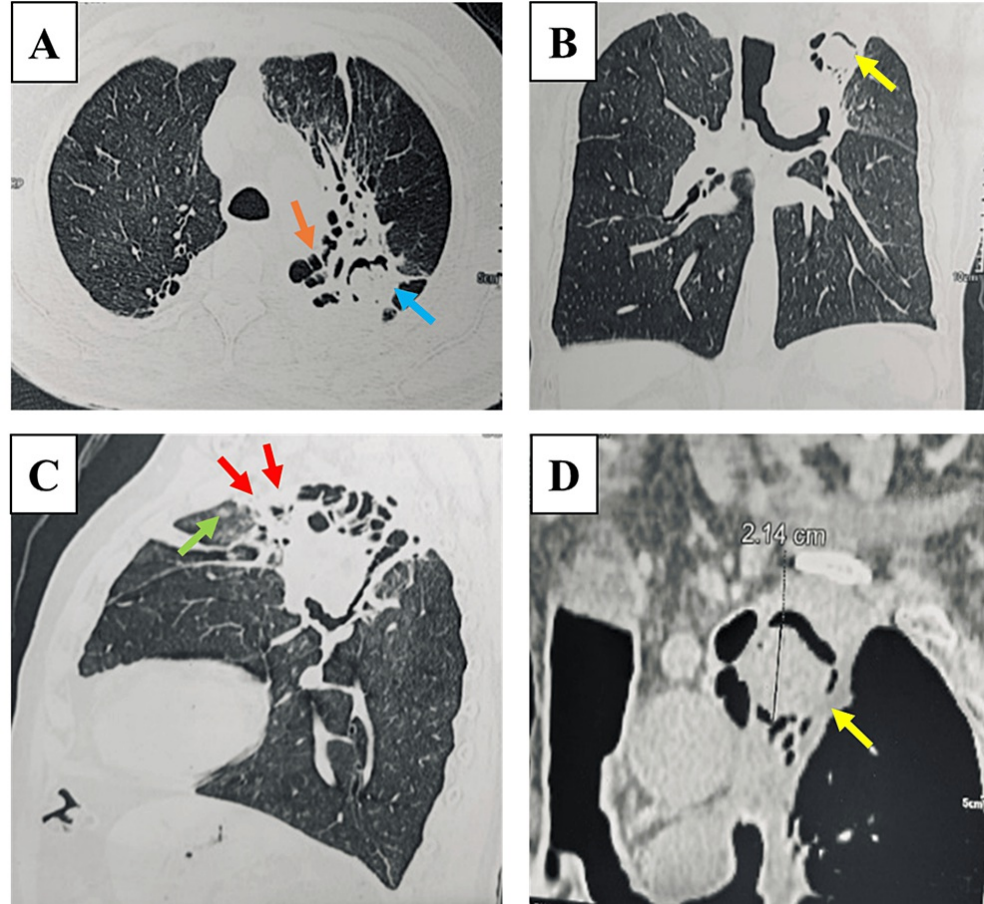
HRCT chest findings A) Lung window in axial section shows left upper lobe fibro-cavity with intracavitary mass (blue arrow) with shaggy cavitary wall and bronchiectasis in atelectatic segments (orange arrow). B) Lung window in coronal section shows air crescent sign (yellow arrow) and bilateral diffuse ground glass opacities. C) Sagittal section shows parenchymal infiltrates (green arrow) and pleural thickening adjacent to the cavity (red arrow). D) Mediastinal window reveals mixed attenuating soft tissue density with an air crescent sign in the left upper lobe (yellow arrow).

A 59-year-old male, non-smoker presented with increased cough and repeated episodes of moderate haemoptysis (60 mL/day) for three days. The patient was diagnosed with pulmonary tuberculosis and treated and cured 10 years ago with 'Category 1' anti-tubercular drugs (six-month regimen), later developing COPD with Bronchiectasis, thereby receiving long-term inhalational bronchodilators and corticosteroids. The patient also had occasional self-limiting episodes of ‘mild’ haemoptysis (~20 mL) for over three years. The patient was negative for viral markers pertinent to HIV, HCV, and HBV. An HRCT chest revealed a left upper lobe cavity with an intracavitary mass demonstrating ‘air-crescent’ sign, parenchymal destruction with peri-broncho-vascular interstitial thickening, dilated bronchi/bronchioles with ipsilateral lung volume reduction along with ground glass opacities, and adjacent pleural thickening (Figure 1). There was no evidence of mediastinal or hilar lymphadenopathy or pleural effusion.

A pulmonary function test showed a ‘moderate’ obstruction with a predicted FEV1 of 67% (GOLD 2024 [2] grading) with no reversibility. *Aspergillus fumigatus*-specific IgG was positive. Bronchoalveolar lavage was negative for bacterial Gram staining, AFB smear, GeneXpert®, conventional bacterial/mycobacterial cultures, and malignant cells but positive for galactomannan (OD=5.41). The presence of one host factor; use of ‘long term’ oral and inhalational corticosteroids, one clinical factor; positive ‘air-crescent’ sign on HRCT chest, and one microbiological factor; and positive BAL galactomannan collectively suggested the diagnosis of ‘probable’ invasive pulmonary aspergillosis as per the diagnosing criteria of EORTC/MSG [3] 2020 (European Organization for Research and Treatment of Cancer and the Mycoses Study Group). However, serum galactomannan was not done.

**Figure 2:**
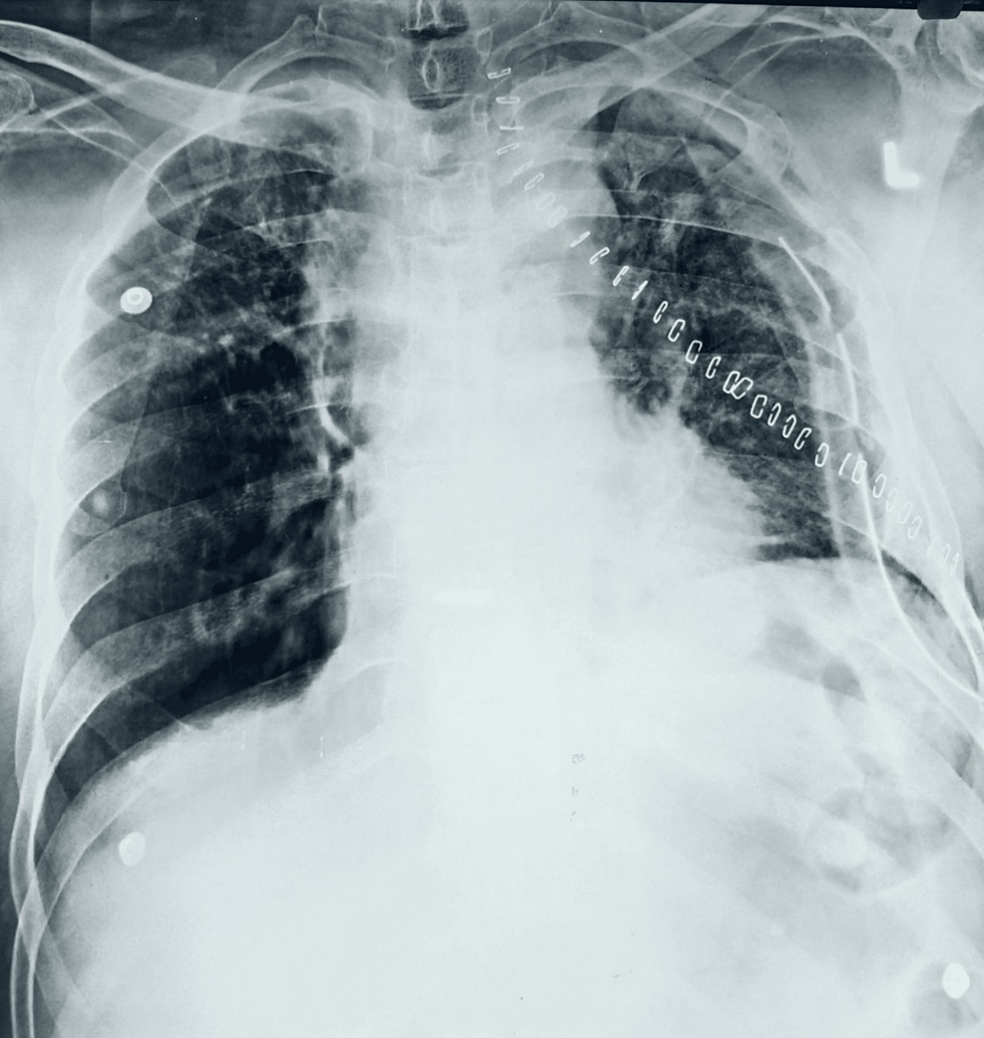
Post-operative chest radiograph Chest X-ray showing adequate left lung expansion following upper lobectomy.

The patient received intravenous voriconazole at a dose of 400 mg (~6 mg/kg), twice daily, following the diagnosis. Haemoptysis was refractory despite the appropriate antifungal therapy, given which the patient was taken up for left upper lobectomy. The post-operative period was uneventful. The patient was given chest physiotherapy, breathing exercises, and incentive spirometry practices. Chest radiography revealed adequate residual lung expansion following left upper lobectomy (Figure 2).

**Figure 3:**
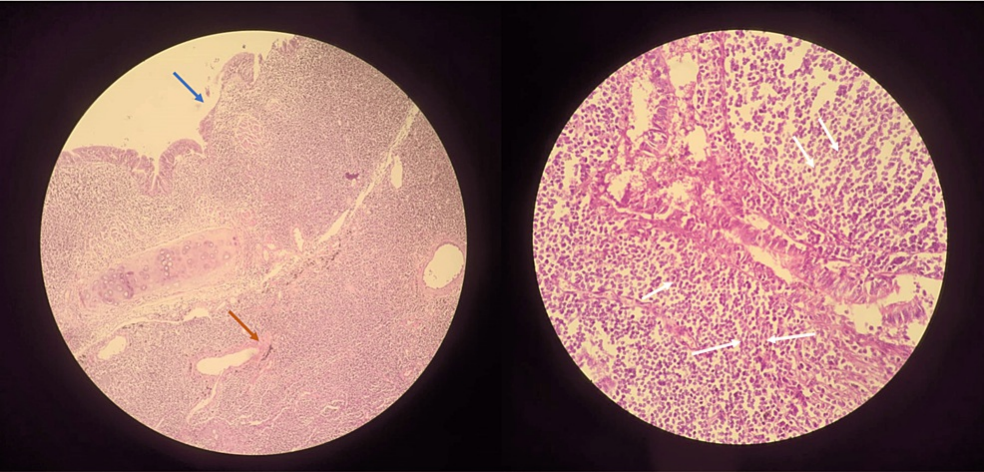
Histopathology of the surgical specimen Figure 3A shows a low-power image of the resected specimen demonstrating destroyed bronchial mucosa (blue arrow) with *Aspergillus *invasion (orange arrow). Figure 3B shows a high-power image of a resected specimen showing extensive monomorphic lymphoid infiltrate in the bronchial wall (white arrows), suggesting lymphoid proliferation.

The surgical specimen tested negative for MTB GeneXpert® and AFB (MGIT) culture but demonstrated *Aspergillus fumigatus* on fungal culture, sensitive to voriconazole (microbroth dilution method). Histopathological examination of the resected lobe showed a markedly dilated and destroyed bronchial wall with septate hyphae invading the bronchial wall and peri-bronchial interstitium (Figure 3A). This confers the diagnosis of ‘proven’ invasive pulmonary aspergillosis. HPE also demonstrated peculiar, dense, diffuse infiltration of monomorphic neoplastic lymphocyte aggregates (Figure 3B) that were positive for both T cell and B cell markers with a Ki-67 proliferation index of 2%-4%, suggesting MALToma (Table 1).

**Table 1:** Findings from the resected left upper lobe

Microbiology	Histopathology	Immunohistochemistry	
Gene Xpert: negative	Dilated, destroyed bronchi with hyphae infiltration into the interstitium	Positive for B cell markers CD20, PAX5, and CD79A and T cell markers CD3 and CD5	Ki 67 proliferation index: 2%-4%
Fungal culture: *Aspergillus fumigatus*	Diffused infiltration of monomorphic neoplastic lymphocytes	Negative for acute lymphoblastic lymphoma: CD10 Others include CD23, CD138, Cyclin D1	
Histopathology and immunohistochemistry suggests – proven Aspergillosis with mucosa-associated lymphoid tissue – lymphoma or MALToma			

**Figure 4:**
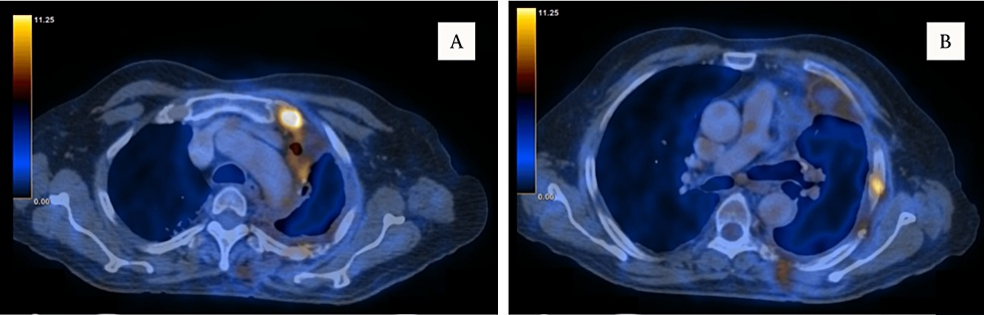
PET-CT scan (post-lobectomy) A) Axial section showing uptake in the surgical area (SUV max: 3.2) B) Axial section showing uptakes in subcarinal node and left ribs

Post-procedure was uneventful, with complete remission of symptoms. PET-CT scan (Figure 4) demonstrated lesions extending from the third to sixth rib, paravertebral, and chest muscles on the left side for which the Tumour Board’s opinion was sought, suggesting chemotherapy. The patient, therefore, received oral chlorambucil (8 mg) at a 14 day-cycle every 28 days. After completion of six cycles of chemotherapy, the patient had complete remission of symptoms with no relapse of haemoptysis and has been on regular follow-up for the past year.

## Discussion

Primary pulmonary lymphoma by itself is a rare entity characterized by the clonal proliferation of lymphoid tissue. Conversely, MALToma is an extremely rare, slow-growing, extranodal lymphoma that develops from memory B cells from the post-germinal centre [4]. The median survival in pulmonary MALToma is close to 10 years [5]. It mostly affects the digestive system, salivary glands, orbit, thyroid gland, and, very infrequently, the lungs. At several instances, the pathological development of MALToma has been found to be associated with long-standing infections, listed in Table 2 [6]. Notably, a tuberculosis antigen-associated chronic inflammation has been often associated with the development of MALToma [7].

Identification of pulmonary MALToma is usually delayed because of nonspecific clinic-radiological characteristics as shown in Table 3 [8-10]. In our case, overlapping radiological features of invasive pulmonary aspergillosis cavity with an intracavitary mass (the air crescent sign) amidst bronchiectasis and fibrosis camouflaged the suspicion of a rare entity like MALToma. Surgical resection was considered instead of conservative interventions like bronchial artery embolization, as resection would be curative for a localized structural lung defect, like in our case. Moreover, procedures like bronchial artery embolization could have temporarily alleviated haemoptysis, masking the presence underlying MALToma, further delaying the diagnosis resulting in dooming complications. The most widely accepted hypothesis for the development of MALToma, anywhere in the body, is because of persistent antigenic stimulation leading to chronic inflammation [11]. In our case, we hypothesize that, chronic inflammation because the presence of Aspergillus antigen could have triggered the development of pulmonary MALToma. To the best of our knowledge, the concurrent existence of MALToma in the setting of aspergillosis has been reported in the literature for the first time. Although MALToma is rare, one should consider it as differentials in scenario of underlying chronic inflammation.

Patients with ‘silent’ MALToma can be conservatively managed just by routine monitoring for clinical or radiological worsening. The mainstay of treatment for localized lymphoma is radiotherapy. In the event of metastasis, chemotherapy using an alkylating agent or a purine analogue with or without an anti-CD20 monoclonal antibody may be employed. The addition of anti-CD20 is superior to chemotherapy alone [12]. Considering long-standing, unresolved haemoptysis, surgical resection was preferred in our case, which paved way for early diagnosis of MALToma, preventing life-threatening complications because of metastasis. Early surgical intervention may be relevant in cases with pre-existing structural lung damage in conjunction with long-standing infections or relapsing haemoptysis.

Clinical pearls: The MALToma group of malignancies are less aggressive malignancies than the usual lymphomas, occurring as a reflect of chronic inflammation. Conditions like invasive pulmonary aspergillosis or other fibro-cavitary disease can masquerade MALToma from the foreground. Surgical intervention should be swiftly considered in cases of persistent haemoptysis with underlying structural lung damage, despite adequate medical management, as it may be curative.

## Conclusions

In conclusion, pulmonary MALToma can be associated in any setting and be possibly triggered by ongoing chronic inflammation. Similarly, in our case of ‘proven’ invasive pulmonary aspergillosis, chronic inflammation triggered by *Aspergillus *antigen could be responsible for the development of MALToma. However, due to a lack of concrete evidence regarding the development of MALToma in such settings, the two entities can be considered independent, as well. The presentation of MALToma is highly variable resulting in substantial delay in diagnosis. Like in our case, MALToma was masqueraded by the presence of invasive pulmonary aspergillosis and was revealed only because of early surgical intervention. Conservative approach in such cases would be detrimental, as early diagnosis and timely management of malignancy would have been impossible. This approach needs further evidence in the form of prospective studies, to evaluate the safety and efficacy of surgical resection over other conservative approaches. Similarly, further molecular studies are warranted to definitively associate the presence of infective antigens (*Aspergillus *antigen, *Mycobacterium *antigen) and the development of MALToma.
